# Supplementary material for: Cryptocentrus steinhardti (Actinopterygii; Gobiidae): a new species of shrimp-goby, and a new invasive to the Mediterranean Sea
Source: PeerJ. 2021 Sep 28;9:e12136. doi: 10.7717/peerj.12136 (PMC8485833; doi:10.7717/peerj.12136)
Supplement: Supplemental Information 1 [file peerj-09-12136-s001.docx]

| **Table S1.** Information for the primers used for PCR and sequencing in this study | | | |
| --- | --- | --- | --- |
| Gene | Primers sequences | Annealing temp (°C) | References |
| *COI* | Fish F2 Forward – 5’-TCGACTAATCATAAAGATATCGGCAC-3’ | 54 | [1] |
|  | Fish F2 Reverse – 5’-ACTTCAGGGTGACCGAAGAATCAGAA-3’ | 54 | [1] |
| *Cytb* | CytbH Forward – 5’-GTGACTTGAAAAACCACCGTTG-3’ | 50 | [2] |
|  | CytbL Reverse – 5’-AATAGGAAGTATCATTCGGGTTTGATG-3’ | 50 | [3] |
| **References**  [1] Ward, R. D., Zemlak, T. S., Innes, B. H., Last, P. R. & Hebert, P. D. N. 2005 DNA barcoding Australia's fish species*. Philosophical Transactions of the Royal Society B: Biological Sciences.* **360**, 1847-1857.  [2] Song, C. B., Near, T. J. & Page, L. M. 1998 Phylogenetic Relations among Percid Fishes as Inferred from Mitochondrial CytochromebDNA Sequence Data*. Mol. Phylogenet. Evol.* **10**, 343-353.  [3] Taberlet, P., Meyer, A. & Bouvet, J. 1992 Unusually large mitochondrial variation in populations of the blue tit *Parus caeruleus. Mol. Ecol.* **1**, 27-36. | | | |
