# Supplementary material for: Cryptocentrus steinhardti (Actinopterygii; Gobiidae): a new species of shrimp-goby, and a new invasive to the Mediterranean Sea
Source: PeerJ. 2021 Sep 28;9:e12136. doi: 10.7717/peerj.12136 (PMC8485833; doi:10.7717/peerj.12136)
Supplement: Supplemental Information 2 [file peerj-09-12136-s002.docx]

| Table S2 – BOLD information for *COI* sequences of all available shrimp-associated gobies used for the phylogenetic analysis in this study (n=111) | | | | |
| --- | --- | --- | --- | --- |
| **Species** | **Sampling locality** | **BOLD accession** | **Barcode Index Number (BIN)** | **Reference** |
| *Cryptocentrus steinhardti* | Mediterranean, Israel | BIM534-17 | N/A | Paratype, this study |
| *Cryptocentrus steinhardti* | Mediterranean, Israel | BIM769-20 | N/A | Holotype, this study |
| *Cryptocentrus steinhardti* | Mediterranean, Israel | BIM770-20 | N/A | Paratype, this study |
| *Cryptocentrus albidorsus* | West Pacific, Taiwan | GBGCA2109-13 | BOLD:ACH4911 | (Thacker et al., 2011) |
| *Cryptocentrus caeruleomaculatus* | Sulawesi, Indonesia | GOBY051-07 | BOLD:AAE9468 | Unpublished |
| *Cryptocentrus caeruleomaculatus* | Sulawesi, Indonesia | GOBY052-07 | BOLD:AAE9468 | Unpublished |
| *Cryptocentrus cebuanus* | South China Sea, Vietnam | GBMNB7330-20 | BOLD:AED2597 | (Thu et al., 2019) |
| *Cryptocentrus cebuanus* | South China Sea, Vietnam | GBMNB7331-20 | BOLD:AED2597 | (Thu et al., 2019) |
| *Cryptocentrus cebuanus* | South China Sea, Vietnam | GBMNB7332-20 | BOLD:AED2597 | (Thu et al., 2019) |
| *Cryptocentrus cinctus* | Pacific Ocean, Australia | GBGCA2101-13 | BOLD:ACH5843 | (Thacker et al., 2011) |
| *Cryptocentrus cinctus* | Pacific Ocean, Australia | GBGCA2102-13 | BOLD:ACH5843 | (Thacker et al., 2011) |
| *Cryptocentrus cinctus* | Pacific Ocean, Papua New Guinea | GBGCA2052-13 | BOLD:ACH5843 | (Thacker et al., 2011) |
| *Cryptocentrus cinctus* | Pacific Ocean, Papua New Guinea | GBGCA2073-13 | BOLD:ACH5843 | (Thacker et al., 2011) |
| *Cryptocentrus cinctus* | Pacific Ocean, Papua New Guinea | GBGCA2084-13 | BOLD:ACH5843 | (Thacker et al., 2011) |
| *Cryptocentrus cinctus* | Pacific Ocean, Papua New Guinea | GBGCA2097-13 | BOLD:ACH5843 | (Thacker et al., 2011) |
| *Cryptocentrus cinctus* | South China Sea, Vietnam | TZAIC190-05 | BOLD:AAC9338 | (Steinke et al., 2009) |
| *Cryptocentrus cinctus* | South China Sea, Vietnam | TZAIC192-05 | BOLD:AAC9338 | (Steinke et al., 2009) |
| *Cryptocentrus cinctus* | South China Sea, Philippines | TZAIC472-06 | BOLD:AAC9338 | (Steinke et al., 2009) |
| *Cryptocentrus cinctus* | South China Sea, Philippines | TZAIC474-06 | BOLD:AAC9338 | (Steinke et al., 2009) |
| *Cryptocentrus cinctus* | South China Sea, Philippines | TZAIC475-06 | BOLD:AAC9338 | (Steinke et al., 2009) |
| *Cryptocentrus cinctus* | Jakarta, Indonesia | TZAIC279-05 | BOLD:AAC9338 | (Steinke et al., 2009) |
| *Cryptocentrus cryptocentrus* | Indian Ocean, Mozambique | DSFSG122-10 | BOLD:AAM4607 | Unpublished |
| *Cryptocentrus cyanotaenia* | Indian Ocean, Sri Lanka | TZAIB080-06 | BOLD:AAR4681 | (Steinke et al., 2009) |
| *Cryptocentrus cyanotaenia* | Indian Ocean, Bangladesh | SAU217-20 | BOLD:AAR4681 | Unpublished |
| *Cryptocentrus cyanotaenia* | Indian Ocean, Bangladesh | GBMNB5992-20 | BOLD:AAR4681 | Unpublished |
| *Cryptocentrus inexplicatus* | Pacific Ocean, Papua New Guinea | GBGCA2074-13 | BOLD:ACH4912 | (Thacker et al., 2011) |
| *Cryptocentrus inexplicatus* | Pacific Ocean, Papua New Guinea | GBGCA2076-13 | BOLD:ACH4912 | (Thacker et al., 2011) |
| *Cryptocentrus inexplicatus* | Pacific Ocean, Papua New Guinea | GBGCA2077-13 | BOLD:ACH4912 | (Thacker et al., 2011) |
| *Cryptocentrus leptocephalus* | Pacific Ocean, Papua New Guinea | GBGCA2051-13 | BOLD:AAB7951 | (Thacker et al., 2011) |
| *Cryptocentrus leptocephalus* | Pacific Ocean, Papua New Guinea | GBGCA2057-13 | BOLD:AAB7951 | (Thacker et al., 2011) |
| *Cryptocentrus leptocephalus* | Pacific Ocean, Papua New Guinea | GBGCA2090-13 | BOLD:AAB7951 | (Thacker et al., 2011) |
| *Cryptocentrus leptocephalus* | Pacific Ocean, Australia | LIFS636-08 | BOLD:AAB7951 | (Steinke et al., 2017) |
| *Cryptocentrus leptocephalus* | Pacific Ocean, Australia | LIFS637-08 | BOLD:AAB7951 | (Steinke et al., 2017) |
| *Cryptocentrus leptocephalus* | Pacific Ocean, Australia | LIFS638-08 | BOLD:AAB7951 | (Steinke et al., 2017) |
| *Cryptocentrus leptocephalus* | Pacific Ocean, Australia | LIFS639-08 | BOLD:AAB7951 | (Steinke et al., 2017) |
| *Cryptocentrus leptocephalus* | Pacific Ocean, Australia | LIFS640-08 | BOLD:AAB7951 | (Steinke et al., 2017) |
| *Cryptocentrus leptocephalus* | Jakarta, Indonesia | TZAIC280-05 | BOLD:AAB7951 | (Steinke et al., 2009) |
| *Cryptocentrus leptocephalus* | Jakarta, Indonesia | TZAIC838-06 | BOLD:AAB7951 | (Steinke et al., 2009) |
| *Cryptocentrus leptocephalus* | Jakarta, Indonesia | TZAIC839-06 | BOLD:AAB7951 | (Steinke et al., 2009) |
| *Cryptocentrus leptocephalus* | Jakarta, Indonesia | TZAIC840-06 | BOLD:AAB7951 | (Steinke et al., 2009) |
| *Cryptocentrus leptocephalus* | Jakarta, Indonesia | TZAIC841-06 | BOLD:AAB7951 | (Steinke et al., 2009) |
| *Cryptocentrus leptocephalus* | South China Sea, Philippines | TZAIC899-06 | BOLD:AAB7951 | (Steinke et al., 2009) |
| *Cryptocentrus leptocephalus* | South China Sea, Philippines | TZAIC900-06 | BOLD:AAB7951 | (Steinke et al., 2009) |
| *Cryptocentrus leptocephalus* | South China Sea, Philippines | TZAIC901-06 | BOLD:AAB7951 | (Steinke et al., 2009) |
| *Cryptocentrus leptocephalus* | South China Sea, Philippines | TZAIC902-06 | BOLD:AAB7951 | (Steinke et al., 2009) |
| *Cryptocentrus leptocephalus* | South China Sea, Philippines | TZAIC903-06 | BOLD:AAB7951 | (Steinke et al., 2009) |
| *Cryptocentrus leptocephalus* | South China Sea, Vietnam | GBMNB7329-20 | BOLD:AAB7951 | (Thu et al., 2019) |
| *Cryptocentrus lutheri* | Indian Ocean, Mozambique | DSFSF035-09 | BOLD:AAJ6520 | Unpublished |
| *Cryptocentrus lutheri* | Indian Ocean, Mozambique | DSFSG181-10 | BOLD:AAJ6520 | Unpublished |
| *Cryptocentrus lutheri* | Persian Gulf, UAE | GBGCA2046-13 | BOLD:AAJ6520 | (Thacker et al., 2011) |
| *Cryptocentrus lutheri* | Persian Gulf, UAE | GBMNB11864-20 | BOLD:AAJ6520 | Unpublished |
| *Cryptocentrus malindiensis* | Indian Ocean, South Africa | DSFSG512-11 | BOLD:AAV9019 | Unpublished |
| *Cryptocentrus maudae* | Indian Ocean, Bangladesh | SAU002-18 | BOLD:ADN9569 | Unpublished |
| *Cryptocentrus maudae* | Indian Ocean, Bangladesh | ANGBF50343-19 | BOLD:ADN9569 | Unpublished |
| *Cryptocentrus nigrocellatus* | West Pacific, Taiwan | GBGCA1963-13 | BOLD:ACH3762 | (Thacker et al., 2011) |
| *Cryptocentrus pavoninoides* | South China Sea, Philippines | TZAIC914-06 | BOLD:AAE4434 | (Steinke et al., 2009) |
| *Cryptocentrus pavoninoides* | South China Sea, Philippines | TZAIC915-06 | BOLD:AAE4434 | (Steinke et al., 2009) |
| *Cryptocentrus pavoninoides* | South China Sea, Philippines | TZAIC916-06 | BOLD:AAE4434 | (Steinke et al., 2009) |
| *Cryptocentrus pavoninoides* | South China Sea, Philippines | TZAIC917-06 | BOLD:AAE4434 | (Steinke et al., 2009) |
| *Cryptocentrus pavoninoides* | South China Sea, Philippines | TZAIC918-06 | BOLD:AAE4434 | (Steinke et al., 2009) |
| *Cryptocentrus* sp. | Pacific Ocean, Papua New Guinea | GBGCA1960-13 | BOLD:ACH3763 | (Thacker et al., 2011) |
| *Cryptocentrus* sp. | Pacific Ocean, Papua New Guinea | GBGCA2083-13 | BOLD:ACH3763 | (Thacker et al., 2011) |
| *Cryptocentrus* sp. | Pacific Ocean, Papua New Guinea | GBGCA2086-13 | BOLD:ACH3763 | (Thacker et al., 2011) |
| *Cryptocentroides arabicus* | Iran, Persian Gulf | GBMNC18033-20 | BOLD:ADW5927 | (Kovačić et al., 2020) |
| *Cryptocentroides arabicus* | Iran, Persian Gulf | GBMNC18034-20 | BOLD:ADW5927 | (Kovačić et al., 2020) |
| *Cryptocentroides arabicus* | Iran, Persian Gulf | GBMNC18035-20 | BOLD:ADW5927 | (Kovačić et al., 2020) |
| *Cryptocentroides arabicus* | Iran, Persian Gulf | GBMNC18036-20 | BOLD:ADW5927 | (Kovačić et al., 2020) |
| *Cryptocentroides arabicus* | Iran, Persian Gulf | GBMNC18037-20 | BOLD:ADW5927 | (Kovačić et al., 2020) |
| *Vanderhorstia mertensi* | Mediterranean, Turkey | GBMIN126292-17 | BOLD:ADL4614 | Unpublished |
| *Amblyeleotris diagonalis* | Red Sea, Saudi Arabia | ANGBF36071-19 | BOLD:AAV9966 | (Isari et al., 2017) |
| *Amblyeleotris diagonalis* | Indian Ocean, Madagascar | SBF191-11 | BOLD:AAV9966 | (Hubert et al., 2015) |
| *Amblyeleotris downingi* | Indian Ocean, Bangladesh | ANGBF56124-19 | BOLD:AEB0217 | Unpublished |
| *Amblyeleotris periophthalma* | Indian Ocean, South Africa | DSFSG557-11 | BOLD:AAU0726 | Unpublished |
| *Amblyeleotris periophthalma* | Indian Ocean, South Africa | DSLAG1772-12 | BOLD:AAU0726 | Unpublished |
| *Amblyeleotris periophthalma* | Indian Ocean, South Africa | DSLAG1776-12 | BOLD:AAU0726 | Unpublished |
| *Ctenogobiops feroculus* | Pacific Ocean, French Polynesia | FPFLB380-12 | BOLD:AAI9996 | (Hubert et al., 2015) |
| *Ctenogobiops feroculus* | Pacific Ocean, French Polynesia | MBFA227-07 | BOLD:AAI9996 | (Hubert et al., 2015) |
| *Ctenogobiops feroculus* | Pacific Ocean, French Polynesia | SCILL311-15 | BOLD:AAI9996 | Unpublished |
| *Ctenogobiops tangaroai* | Pacific Ocean, Wallis and Futuna | FUT022-18 | BOLD:ADT4401 | Unpublished |
| *Mahidolia mystacina* | Indo-Pacific Ocean | GBGCA2093-13 | BOLD:ACH4692 | (Thacker et al., 2011) |
| *Mahidolia* sp. | Red Sea, Saudi Arabia | ANGBF36367-19 | BOLD:ADC2350 | (Isari et al., 2017) |
| *Mahidolia* sp. | Red Sea, Saudi Arabia | ANGBF36368-19 | BOLD:ADC2350 | (Isari et al., 2017) |
| *Mahidolia* sp. | Red Sea, Saudi Arabia | GBMIN97273-17 | BOLD:ADC2350 | (Isari et al., 2017) |
| *Stonogobiops nematodes* | Indian Ocean, South Africa | DSFSG999-13 | BOLD:ACH3076 | Unpublished |
| *Stonogobiops medon* | Pacific Ocean, French Polynesia | MARQ008-12 | BOLD:ACD1971 | (Hubert et al., 2015) |
| *Stonogobiops medon* | Pacific Ocean, French Polynesia | MARQ009-12 | BOLD:ACD1971 | (Hubert et al., 2015) |
| *Stonogobiops medon* | Pacific Ocean, French Polynesia | MARQ295-12 | BOLD:ACD1971 | (Hubert et al., 2015) |
| *Stonogobiops medon* | Pacific Ocean, French Polynesia | MOH105-16 | BOLD:ACD1971 | (Hubert et al., 2015) |
| *Stonogobiops xanthorhinica* | Pacific Ocean | GBGCA2095-13 | BOLD:ACH6364 | (Thacker et al., 2011) |
| *Psilogobius mainlandi* | Pacific Ocean, Hawaii | KANB050-20 | BOLD:ADJ0893 | Unpublished |
| *Psilogobius mainlandi* | Pacific Ocean, Hawaii | KANB256-20 | BOLD:ADJ0893 | Unpublished |
| *Psilogobius mainlandi* | Pacific Ocean, Hawaii | KANB263-20 | BOLD:ADJ0893 | Unpublished |
| *Myersina filifer* | Pacific Ocean, Japan | ABFJ165-06 | BOLD:AAR4680 | (Zhang & Hanner, 2011) |
| *Myersina filifer* | West Pacific, Taiwan | ANGBF36369-19 | BOLD:AAR4680 | Unpublished |
| *Myersina filifer* | West Pacific, China | ANGBF36370-19 | BOLD:AAR4680 | Unpublished |
| *Myersina filifer* | West Pacific, China | GBMIN120959-17 | BOLD:AAR4680 | Unpublished |
| *Myersina filifer* | South China Sea, China | SCS1041-16 | BOLD:AAR4680 | Unpublished |
| *Myersina filifer* | South China Sea, China | SCS1042-16 | BOLD:AAR4680 | Unpublished |
| *Myersina filifer* | South China Sea, China | SCS1043-16 | BOLD:AAR4680 | Unpublished |
| *Myersina filifer* | South China Sea, China | SCS1044-16 | BOLD:AAR4680 | Unpublished |
| *Myersina filifer* | South China Sea, China | SCS1045-16 | BOLD:AAR4680 | Unpublished |
| *Myersina filifer* | South China Sea, China | SCS1046-16 | BOLD:AAR4680 | Unpublished |
| *Lotilia* sp. | Red Sea, Saudi Arabia | ANGBF27845-19 | BOLD:ADK6708 | (Troyer et al., 2018) |
| *Lotilia* sp. | Red Sea, Saudi Arabia | ANGBF27846-19 | BOLD:ADK6708 | (Troyer et al., 2018) |
| *Lotilia* sp. | Red Sea, Saudi Arabia | GBMIN97272-17 | BOLD:ADK6708 | (Isari et al., 2017) |
| *Lotilia* sp. | Red Sea, Saudi Arabia | GBMIN121883-17 | BOLD:ADK6708 | (Isari et al., 2017) |
| *Lotilia* sp. | Red Sea, Saudi Arabia | GBMIN126680-17 | BOLD:ADK6708 | (Isari et al., 2017) |
| *Lotilia* sp. | Red Sea, Saudi Arabia | GBMIN126702-17 | BOLD:ADK6708 | (Isari et al., 2017) |
| *Lotilia* sp. | Red Sea, Saudi Arabia | GBMIN132458-17 | BOLD:ADK6708 | (Isari et al., 2017) |
| *Tomiyamichthys lanceolatus* | Indo-Pacific Ocean | GBGCA2089-13 | BOLD:ACI1885 | (Thacker et al., 2011) |
| *Gobius niger* (Outgroup) | Mediterranean, Israel | BIM053-13 | BOLD:AAC0219 | Unpublished |

References

Hubert, N., Espiau, B., Meyer, C., & Planes, S. (2015). Identifying the ichthyoplankton of a coral reef using DNA barcodes. *Molecular Ecology Resources*, *15*(1), 57–67.

Isari, S., Pearman, J. K., Casas, L., Michell, C. T., Curdia, J., Berumen, M. L., & Irigoien, X. (2017). Exploring the larval fish community of the central Red Sea with an integrated morphological and molecular approach. *PLoS ONE*. https://doi.org/10.1371/journal.pone.0182503

Kovačić, M., Sadeghi, R., & Esmaeili, H.-R. (2020). New species of Silhouettea (Teleostei: Gobiidae) from Qeshm Island, Iran and the DNA barcoding of the Persian Gulf and Oman Sea gobies.‏. *Zootaxa*, *4750*(1), 49–66. https://europepmc.org/article/med/32230052

Steinke, D., de Waard, J. R., Gomon, M. F., Johnson, J. W., Larson, H. K., Lucanus, O., Moore, G. I., Reader, S., & Ward, R. D. (2017). DNA barcoding the fishes of Lizard island (Great Barrier Reef). *Biodiversity Data Journal*. https://doi.org/10.3897/BDJ.5.e12409

Steinke, D., Zemlak, T. S., & Hebert, P. D. N. (2009). Barcoding nemo: DNA-based identifications for the ornamental fish trade. *PLoS ONE*. https://doi.org/10.1371/journal.pone.0006300

Thacker, C. E., Thompson, A. R., & Roje, D. M. (2011). Phylogeny and evolution of Indo-Pacific shrimp-associated gobies (Gobiiformes: Gobiidae). *Molecular Phylogenetics and Evolution*, *59*(1), 168–176. https://doi.org/10.1016/j.ympev.2011.02.007

Thu, P. T., Huang, W. C., Chou, T. K., Van Quan, N., Van Chien, P., Li, F., Shao, K. T., & Liao, T. Y. (2019). DNA barcoding of coastal ray-finned fishes in Vietnam. *PLoS ONE*. https://doi.org/10.1371/journal.pone.0222631

Troyer, E. M., Coker, D. J., & Berumen, M. L. (2018). Comparison of cryptobenthic reef fish communities among microhabitats in the Red Sea. *PeerJ*. https://doi.org/10.7717/peerj.5014

Zhang, J.-B., & Hanner, R. (2011). DNA barcoding is a useful tool for the identification of marine fishes from Japan. *Biochemical Systematics and Ecology*, *39*(1), 31–42. http://www.sciencedirect.com/science/article/pii/S0305197810002292
